# The Challenges of Diagnosing Familial Dysbetalipoproteinemia: A Case Associated With a Rare ApoE Variant

**DOI:** 10.1155/carm/8758502

**Published:** 2026-03-02

**Authors:** Spencer Rowland, Kent Brummel, Rajani Aatre, Eric J. Brandt

**Affiliations:** ^1^ Cardiovascular Medicine Department, University of Michigan, Ann Arbor, Michigan, USA, umich.edu

**Keywords:** apolipoprotein E, dysbetalipoproteinemia, hypertriglyceridemia

## Abstract

Familial dysbetalipoproteinemia (FDB) is a lipid disorder characterized by defective clearance of triglyceride‐rich lipoprotein remnants. Definitive diagnosis has relied on genetic markers, lipid profiles, and specialized lipid assays including gel electrophoresis that demonstrates the characteristic beta‐band consistent with enriched small VLDL and IDL. We present a case of a 51‐year‐old female with progressive hyperlipidemia despite a stable plant‐based diet and regular exercise. Her lipid profile met many of the diagnostic criteria for FDB (ApoB < 120 mgd/L, TG > 133 mg/dL [1.5 mmol/L], and TG/ApoB ratio < 8.8). However, advanced lipid testing failed to demonstrate hallmark lipid remnant accumulation, likely due to statin therapy initiation prior to the time of testing. Genetic testing revealed heterozygosity for the ApoE2 variant (Arg176Cys) and another novel variant of unknown significance (VUS), 593 G > A (Arg198His), on the same allele (herein termed ApoE2‐Wolverine). The ApoE2‐Wolverine variant may be contributing to the patient’s dyslipidemia; however, further investigation into its functional significance and cardiovascular implications is needed. Her treatment with rosuvastatin 10 mg, 2 g of daily eicosapentaenoic acid (EPA), and lifestyle modifications contributed to improvements in her lipid levels. This case highlights the diagnostic challenges in FDB, especially when novel genetic variants are involved. While many criteria for FDB were met, confirmatory gel electrophoresis and genetic testing were inconclusive. This case underscores the need for multimodal assessment in FDB diagnosis, incorporating genetic analysis, lipid profiles, and therapeutic response.

## 1. Introduction

ApoE encodes apolipoprotein E (ApoE), a glycoprotein on triglyceride‐rich lipoproteins like VLDL, IDL, and chylomicrons. ApoE binds to LDL receptors and heparin sulfate proteoglycans (HSPs), facilitating lipoprotein clearance and triggering triglyceride hydrolysis [1]. Reduced ApoE​ binding efficiency increases IDL and VLDL levels in the bloodstream [2].

Familial dysbetalipoproteinemia (FDB) (i.e., classic Fredrickson Type 3 hyperlipidemia) typically occurs with homozygosity of ApoE2 (Arg176Cys), which accounts for 90% of cases [3]. Despite the ApoE2 variant not being in the binding domain, it is theorized that this change in charge (losing a positive charge from arginine) leads to protein misfolding, which reduces the binding affinity of ApoE for the LDL receptor and HSP proteins.

FDB patients exhibit elevated remnant lipoproteins and triglycerides, leading to lipid deposits in the skin. These manifest as cutaneous xanthomas, including palmar crease xanthomas, tuberous or tendinous xanthomas, and xanthelasma [4, 5]. Lipid profiles suggestive of FDB include ApoB < 120 mgd/L, TG > 133 mg/dL (1.5 mmol/L), TG/ApoB ratio < 8.8, and TC:ApoB > 2.4 mg/dL [6].

Homozygous ApoE2 carriers show incomplete penetrance, indicating that some function of this pathway (or others) remains intact [2]. Phenotypic expression of dysbetalipoproteinemia typically manifests in conjunction with the development of insulin resistance, menopause (loss of estrogen’s protective effect), obesity, diabetes mellitus, alcohol abuse, and/or hypothyroidism [3].

Diagnosing FDB is important because it identifies patients at markedly increased risk for premature atherosclerotic cardiovascular disease due to lifelong elevations in remnant lipoproteins. While treatment still centers on lifestyle modification and lipid‐lowering therapy, an accurate diagnosis prompts more aggressive lipid‐lowering goals, earlier initiation of therapy, and closer monitoring of triglyceride‐rich lipoproteins. It also guides screening of family members when appropriate, helping identify others who may carry ApoE variants and benefit from preventive care.

## 2. Case Presentation

A 51‐year‐old female with a history of acne and prediabetes was referred to the lipidology clinic in June 2022 for hyperlipidemia. Her cholesterol and triglyceride levels had increased progressively from January 2020 to March 2022 (Table 1). She reported no recent changes in medications, lifestyle, or diet, except for entering menopause over a year earlier. She was using topical tretinoin cream 0.05% for 30 years but had no other prescribed medications.

**TABLE 1:** Patient lipid and hemoglobin a1c values and notable events.

Notable events	Date	Total cholesterol (mg/dL)	HDL‐C (mg/dL)	TG (mg/dL)	LDL‐C (mg/dL)	Non–HDL‐C (mg/dL)	ApoB or Lp (a) (mg/dL)	Hemoglobin a1c
	2/15/2012	180	43	127	111	137		
	6/8/2013	184	39	104	124	145		
	1/29/2020	242	51	140	163	191	ApoB: 112	5.1%
4/24 = LMP	4/23/2021	325	45	339	212	280		5.3%
	3/18/2022	339	44	412	155 (∗ dLDL)	295		5.4%
Therapy initiated	6/10/2022	298	46	384	176	253	Lp (a): 45	5.8%
	8/5/2022	169	45	172	90	124	ApoB: 86	5.7%
Resumed tretinoin 10/2022	12/20/2022	150	49	92	82	101		
Not fasting	2/1/2023	159	45	185	77	114		5.7%
Mayo Clinic assay	3/27/2023	143	40	166	66			
	8/25/2023	179	48	177	96	131		
	11/15/2023	158	53	81	89	105	Apo B: 80	

Her dietary pattern was primarily whole food, plant‐based with no added salt in cooking and had not changed during the period of lipid elevation. She exercised daily, achieving at least 10,000 steps through 30 min of jogging and at least 30–60 min of walking. She reported no family history of hyperlipidemia or hypertriglyceridemia. Initial physical examination revealed no xanthelasma, palmar xanthomas, or other significant findings.

Table 1 contains her lipid levels at presentation. Given the concern for familial hyperlipidemia or FDB, genetic testing was sent to GB HealthWatch (Dyslipidemia and ASCVD Comprehensive Panel, San Diego, CA). Genetic testing demonstrated the ApoE2 variant (Arg176Cys) and another novel variant of unknown significance (VUS), 593 G> A (Arg198His), on the same allele (herein termed ApoE2‐Wolverine). This VUS had not previously been reported in dbSNP, UK Biobank, or gnomAD. Her other allele was a normal ApoE3 making her heterozygous ApoE2/E3. Additional findings included in her genetics report were identified as risk alleles an APOA5 ∗ 158C > T single‐nucleotide polymorphism (SNP), CETP 1403G > A (Arg468Gln), SOD3 691C > G (Arg231Gly), FTO 46–43098T > C, SH2B1 1450A > G (Thr484Ala), and SLCO1B1 521T > C (Val174Ala).

Given the patient’s lipid laboratory values and genetic testing, a diagnosis of FDB was considered. She was initiated on rosuvastatin 10 mg and advised to increase strenuous exercise to 40 min daily four times per week. This, combined with a refocus on ideal dietary habits, led to significant improvements in her lipid profile by December 2022 (cholesterol 150 mg/dL, HDL‐C 49 mg/dL, TG 92 mg/dL, LDL‐C 82 mg/dL, and non–HDL‐C 101 mg/dL) (Table 1).

To confirm the diagnosis of FDB, specialized laboratory testing was deemed necessary. Consequently, in February 2023, the patient was given a brief respite from her stringent dietary regimen, which resulted in a slight increase in her lipid levels (Table 1). Subsequently, an advanced lipid metabolic profile was sent to and conducted at the Mayo Clinic. However, this assay failed to detect the typical lipid remnant accumulation associated with dysbetalipoproteinemia.

Her peak lipid values, however, in addition to her genetics, meet some criteria for a diagnosis of dysbetalipoproteinemia. For example, she has an ApoB less than 120 mgd/L, triglycerides greater than 133 mg/dL (1.5 mmol/L), a triglyceride/ApoB ratio less than 8.8, although we did not capture a TC:ApoB of greater than 2.4 mg/dL [6]. By this algorithm, she would meet the criteria for FDB if chylomicron and VLDL remnants were detected on gel electrophoresis.

In this patient case, evaluation for FDB did not yield a definitive diagnosis. The advanced lipid metabolic panel was nondiagnostic, and genetic testing identified an ApoE2 allele with a VUS (Arg198His). A polygenic or oligogenic dyslipidemia is also possible. Nevertheless, the patient’s lipid profile and their favorable response to statin therapy and lifestyle modification are consistent with features described in FDB. While alternative explanations cannot be excluded, this case illustrates a presentation that could represent FDB and highlights the diagnostic challenges associated with atypical or uncertain ApoE variants. For continued management, our patient remained on rosuvastatin 10 mg. However, in July 2022, she was advised to discontinue tretinoin due to the known potential of oral tretinoin to elevate triglyceride levels, although the association with topical tretinoin specifically is less clear [7, 8]. She resumed tretinoin use in October 2022 on her own. However, no elevations in her lipid levels were observed thereafter (Table 1).

Upon her return to the clinic in August 2023, despite adhering to increased exercise and a stable diet, her lipid levels showed a slight elevation from previous measures (cholesterol 179 mg/dL, HDL‐C 48 mg/dL, TG 177 mg/dL, LDL‐C 96 mg/dL, and non–HDL‐C 131 mg/dL). Additionally, she reported experiencing more menopausal symptoms. She was initiated on over‐the‐counter EPA 2 g daily, which resulted in improved lipid levels (Table 1). At this visit, a repeat examination also revealed possible xanthomas on her posterior distal phalange joints (Figure 1).

FIGURE 1Distal digit xanthomas on the 5th digit, 09/2023.

**Figure:**
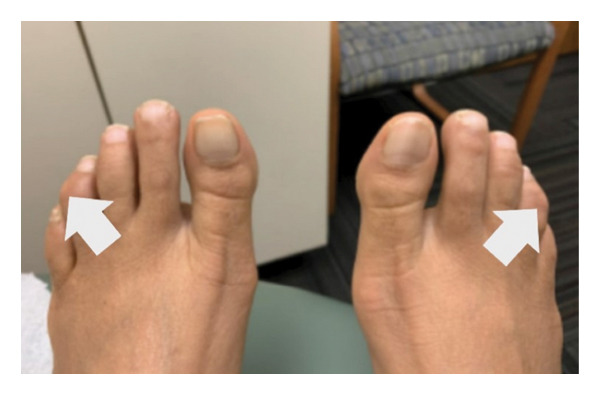
(a)

**Figure:**
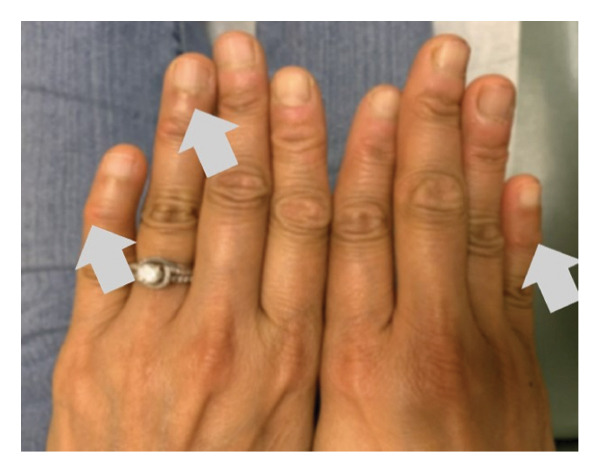
(b)

## 3. Discussion

We present a notable case of severe hyperlipidemia and hypertriglyceridemia in a patient with an ApoE2 variant and a VUS occurring on the same allele (ApoE2‐Wolverine). This case highlights the challenges inherent in diagnosing FDB.

The diagnostic criteria for FDB remain varied, reflecting the difficulty of establishing a consensus. The algorithm proposed by Sniderman et al., validated using ultracentrifugation, successfully identifies individuals with FDB through TG and ApoB levels [6]. However, despite meeting this algorithm’s criteria, our patient could not be definitively diagnosed due to the absence of characteristic findings on gel electrophoresis, although testing was not done with peak lipids. Ultracentrifugation and gel electrophoresis remain the diagnostic gold standards for FDB. Gel electrophoresis detects cholesterol‐enriched VLDL at the β‐position and has high specificity in untreated patients [9]. However, our patient’s lipid levels were significantly reduced after nearly 9 months of statin and dietary therapy, potentially diminishing gel electrophoresis sensitivity by lowering remnant protein levels. We hypothesize that our patient did not have significant enough levels of remnant protein to be detected on gel electrophoresis due to her adequate management and significantly reduced lipid levels. Notably, pharmacological therapy for FDB often involves statins and fibrates. When rosuvastatin proved insufficient, we initiated EPA 2 g daily, aiming to reduce VLDL and remnant accumulation. This adjustment improved her lipid panel [10, 11].

A review of the current literature reveals only one other reported case of a variant affecting the same amino acid. The family with this variant had an arginine to cysteine substitution at the first position of Codon 180 in the hinge region of the ApoE protein. The variant was first identified in a 42‐year‐old woman who presented with hypertriglyceridemia [12]. It was found that the variant altered the recognition site for endonuclease HaeII, leading to the proposal that the variant likely caused hypertriglyceridemia through the inhibition of triglyceride hydrolysis associated with VLDL [12]. While this is the only variant at this site reported, other rare ApoE variants have been linked to FDB and hypertriglyceridemia [13].

Our patient’s two variants on the same ApoE allele are noteworthy. Carriers of a single ApoE2 allele typically do not develop lipid disorders, but autosomal dominant variants account for ∼10% of FDB cases. Such cases exhibit similar atherogenic lipid profiles and respond well to standard therapies [14]. Some other rare cis variants, such as ApoE‐4 Philadelphia (Glu13Lys, Arg145Cys), APO1‐Hammersmith (Lys146Asn, Arg147Trp), ApoE1 (Gly127Asp, Arg158Cys), ApoE3 (Arg112Cys, Cys142Arg), and ApoE7 (Glu244Lys, Glu245Lys), have been described in the literature and linked to phenotypes similar to that of autosomal dominant FDB, highlighting the pathogenic potential of such rare cis variants [14, 15]. Accordingly, this case may represent an autosomal dominant form of FDB associated with a VUS. However, it is also possible that the patient’s mixed dyslipidemia reflects an underlying polygenic or oligogenic etiology rather than being driven by a single causative variant. Notably, our patient lacked a family history of lipid disorders, and other family members declined genetic and lipid testing which further limited our ability to definitively diagnose FDB and establish inheritance of the allele.

Another crucial consideration in the case is our patient’s APOA5 c ∗ 158C > T SNP. Prior studies indicate that it may be a risk factor for developing dyslipidemia and moderate to severe hypertriglyceridemia. Furthermore, multiple APOA5‐associated SNPs and genetic variants have been identified and associated with FDB and hypertriglyceridemia [16, 17].

In this case, the presence of a VUS in ApoE alongside a lipid profile closely resembling FDB underscores the importance of documenting such presentations to help refine diagnostic criteria. The case also highlights the complexity of FDB diagnosis and exemplifies key considerations in its management including statins, EPA, and lifestyle interventions.

## Author Contributions

S.R. drafted the manuscript and performed background research. K.B. and E.J.B. provided patient care and conducted genetic variant research. R.A. provided consultation on genetic variants.

## Funding

No funding was received for this manuscript.

## Disclosure

All authors approved the final manuscript.

## Consent

Written informed consent was obtained from the patient for publication of this case report and all accompanying clinical information and images.

## Conflicts of Interest

The authors declare no conflicts of interest.

## Data Availability

All data underlying this case report are available to the journal and its reviewers upon request.

## References

[1] Davidson M. H. , Toth P. P. , and Maki K. C. , Therapeutic Lipidology, 2021, Springer International Publishing, 10.1007/978-3-030-56514-5.

[2] Mahley R. , Huang Y. , and Rall S. , Pathogenesis of Type III Hyperlipoproteinemia (Dysbetalipoproteinemia): Questions, Quandries, and Paradoxes, Journal of Lipid Research. (1999) 40, no. 11, 1933–1949, 10.1016/S0022-2275(20)32417-2. 10552997

[3] Smelt A. and de Beer F. , Apolipoprotein E and Familial Dysbetalipoproteinemia: Clinical, Biochemical, and Genetic Aspects, Seminars in Vascular Medicine. (2004) 4, no. 3, 249–257, 10.1055/s-2004-861492, 2-s2.0-11844286928. 15630634

[4] Koopal C. , Marais A. D. , and Visseren F. L. J. , Familial Dysbetalipoproteinemia: An Underdiagnosed Lipid Disorder, Current Opinion in Endocrinology Diabetes and Obesity. (2017) 24, no. 2, 133–139, 10.1097/MED.0000000000000316, 2-s2.0-85009830707. 28098593

[5] Rothschild M. , Duhon G. , Riaz R. et al., Pathognomonic Palmar Crease Xanthomas of Apolipoprotein E2 Homozygosity Familial Dysbetalipoproteinemia, JAMA Dermatology. (2016) 152, no. 11, 1275–1276, 10.1001/jamadermatol.2016.2223, 2-s2.0-84997784894. 27603268

[6] Sniderman A. D. , De Graaf J. , Thanassoulis G. , Tremblay A. J. , Martin S. S. , and Couture P. , The Spectrum of Type III Hyperlipoproteinemia, Journal of Clinical Lipidology. (2018) 12, no. 6, 1383–1389, 10.1016/j.jacl.2018.09.006, 2-s2.0-85054560770. 30318453

[7] Gerber L. and Erdman J. , Retinoic Acid and Hypertriglyceridemia, Annals of the New York Academy of Sciences. (1981) 359, no. 1, 391–392, 10.1111/j.1749-6632.1981.tb12766.x, 2-s2.0-0019391004. 6942686

[8] Melnik B. , Bros U. , and Plewig G. , Evaluation of the Atherogenic Risk of Isotretinoin-Induced and Etretinate-Induced Alterations of Lipoprotein Cholesterol Metabolism, Journal of Investigative Dermatology. (1987) 88, no. 3, 39–43, 10.1159/000248880, 2-s2.0-0023628639. 3469279

[9] Blom D. J. , Byrnes P. , Jones S. , and Marais A. D. , Non-Denaturing Polyacrylamide Gradient Gel Electrophoresis for the Diagnosis of Dysbetalipoproteinemia, Journal of Lipid Research. (2003) 44, no. 1, 212–217, 10.1194/jlr.D200013-JLR200, 2-s2.0-0037237989. 12518040

[10] Buckley R. , Shewring B. , Turner R. , Yaqoob P. , and Minihane A. M. , Circulating Triacylglycerol and ApoE Levels in Response to EPA and Docosahexaenoic Acid Supplementation in Adult Human Subjects, British Journal of Nutrition. (2004) 92, no. 3, 477–483, 10.1079/BJN20041235, 2-s2.0-6944255823. 15469651

[11] Dallongeville J. , Boulet L. , Davignon J. , and Lussier-Cacan S. , Fish Oil Supplementation Reduces beta-very Low Density Lipoprotein in Type III Dysbetalipoproteinemia, Arteriosclerosis, Thrombosis, and Vascular Biology. (1991) 11, no. 4, 864–871, 10.1161/01.atv.11.4.864. 2065040

[12] Hoffmann M. M. , Scharnagl H. , Köster W. , Winkler K. , Wieland H. , and März W. , Apolipoprotein E1 Baden (Arg180⟶Cys), Clinica Chimica Acta. (2001) 303, no. 1, 41–48, 10.1016/S0009-8981(00)00372-7, 2-s2.0-0035144668. 11163021

[13] van den Maagdenberg A. M. , Weng W. , de Bruijn I. H. et al., Characterization of Five New Mutants in the carboxyl-terminal Domain of Human Apolipoprotein E: No Cosegregation with Severe Hyperlipidemia, The American Journal of Human Genetics. (1993) 52, no. 5, 937–946, 10.1016/S0002-9297(07)62438-2. 8488843 PMC1682049

[14] Koopal C. , Marais A. D. , Westerink J. , and Visseren F. L. J. , Autosomal Dominant Familial Dysbetalipoproteinemia: A Pathophysiological Framework and Practical Approach to Diagnosis and Therapy, Journal of Clinical Lipidology. (2017) 11, no. 1, 12–23.e1, 10.1016/j.jacl.2016.10.001, 2-s2.0-85006137493. 28391878

[15] Richard P. , Beucler I. , Pascual De Zulueta M. , Biteau N. , De Gennes J. L. , and Iron A. , Compound Heterozygote for Both Rare Apolipoprotein E1 (Gly127—>Asp, Arg158—>Cys) and E3(Cys112—>Arg, Arg251—>Gly) Alleles in a Multigeneration Pedigree With Hyperlipoproteinaemia, Clinical Science. (1997) 93, no. 1, 89–95, 10.1042/cs0930089, 2-s2.0-0030860846. 9279208

[16] Jacob J. , Boczkowska S. , Zaluska W. , and Buraczynska M. , Apolipoprotein A5 Gene Polymorphism (Rs662799) and Cardiovascular Disease in End-Stage Kidney Disease Patients, BMC Nephrology. (2022) 23, no. 1, 10.1186/s12882-022-02925-1. PMC9450442 36071387

[17] Satny M. , Todorovova V. , Altschmiedova T. et al., Genetic Risk Score in Patients With the ApoE2/E2 Genotype as a Predictor of Familial Dysbetalipoproteinemia, Journal of Clinical Lipidology. (2024) 18, no. 2, e230–e237, 10.1016/j.jacl.2023.11.010. 38044203

